# Predictors of independence in older people: A longitudinal, population-based study using the CARE75 + cohort

**DOI:** 10.1186/s12877-025-05927-4

**Published:** 2025-04-28

**Authors:** Emily Taylor, Victoria A Goodwin, Andrew Clegg, Lesley Brown, Julia Frost, Susan Ball

**Affiliations:** 1 Faculty of Health and Life Sciences, University of Exeter, Exeter, UK; 2 Academic Unit for Ageing and Stroke Research, University of Leeds, Leeds, UK; 3 Academic Unit for Ageing and Stroke Research, Bradford Teaching Hospitals NHS Foundation Trust, Bradford, UK; 4 NIHR Applied Research Collaboration South West Peninsula, University of Exeter, Exeter, UK

**Keywords:** Activities of daily living, Independence, Older people, Predictors, Community-dwelling

## Abstract

**Background:**

The ability to predict older people’s functional independence has implications for the development and provision of services to improve individual sense of self and wellbeing.

**Methods:**

Using linear regression analyses we identified predictors of independence, measured using the Nottingham Extended Activities of Daily Living (NEADL) scale, at 12 and 24-months from baseline. Data were obtained from 1277 community-dwelling people aged ≥ 75. Multivariable models included predictors that were selected through review of existing literature, perspectives of older people, and univariable analyses. Multiple imputation was used to account for missing data.

**Results:**

Participants’ mean age was 84.61 years (SD 4.95) and just over half were female (*n* = 655, 51.29%). At baseline, participants had a mean NEADL score of 53.82 (SD 13.19). Younger age, fewer hours of informal help received, no registered visual impairment, lower frailty, living alone, higher cognitive function, greater physical function, absence of depression, and higher baseline NEADL were significant predictors of greater independence at 12-months. Younger age, higher baseline NEADL score, living alone, less frailty, higher cognitive function, alcohol consumption, greater physical function, and absence of depression predicted greater independence at 24-months.

**Conclusion:**

Depression and frailty are important predictors of an older person’s independence with other variables such as activities of daily living, age, cognitive function, alcohol consumption, and living status also having an impact over a prolonged period. Refining understanding of the mechanisms within frailty and depression is likely to improve targeting of support and interventions, which will have a lasting impact on older people’s independence.

**Supplementary Information:**

The online version contains supplementary material available at 10.1186/s12877-025-05927-4.

## Introduction

Being functionally independent contributes to greater self-esteem, wellbeing and quality of life, especially in the context of older age, and may mediate the impact of ill-health and physical impairment [1–4]. Independence is therefore an important concept in society where healthy life expectancy has not kept pace with total life expectancy leading to a greater number of people living for longer in poorer health [5]. In health and social care, independence is a common treatment goal both because it is valuable to the individual, and because independent self-management of health conditions and instrumental activities of daily living can reduce the resources required from strained services [6–9]. Identifying predictors of independence can inform the optimal use of resources to help older people improve, achieve, and maintain control and agency in later life.

Existing work identifying predictors of independence mainly focus on return to independence following an adverse event, such as when recovering from an acute illness and engaging in a rehabilitation program [10, 11]. Predictors (such as age, gender, morbidity, and level of physical activity) have predominantly been selected based on clinical and academic models of independence without input from older people [12, 13]. Further, there has been much inconsistency in the breadth and type of covariates included in existing statistical analyses of the predictors of independence making it difficult to interpret the independent impact of a given predictor [12, 13]. As part of an integrated mixed methods project, the authors of this study utilise the findings of a preceding qualitative study [14] in which a cohort of community-dwelling older people were asked about what they believed were the most important predictors of independence.

Identifying predictors that could indicate a threat to independence before an adverse event has taken place could inform strategies for more preventative care. Predictors rarely impact an outcome without influence from confounding variables. At all ages, but especially in old age, multiple and complex factors intersect to impact a person’s lived experience. A broader range of predictors that are informed by older people’s perspectives on what is important for independence is required to better represent and understand the complexity of influences on independence for community-dwelling older people aged 75+.

The aim of this study was to identify predictors of independence over time. The main analysis identified predictors at 12 months from baseline and a secondary analysis identified predictors at24 months from baseline to explore their ability to predict change over a longer time period.

## Methods

### Study setting and participants

Data for this study were obtained from an existing longitudinal cohort study, the Community Ageing Research 75+ (CARE75+) cohort [15]. The cohort study design collects data on individuals at regular intervals over a period of 4 years, enabling the exploration of changes over time and the generation of information that may help to predict change. CARE75 + is an open and ongoing cohort study (Trial Registration Number: ISRCTN16588124). Physical, psychosocial and cognitive assessments are conducted with participants by trained research staff at baseline, 6, 12, 24 and 48 months. The data analysed and reported in this paper were collected between January 2015 and April 2021, from 7 sites across England by research staff from the CARE75 + cohort study. CARE75 + study design and data collection procedures are reported in detail elsewhere [15].

CARE75 + inclusion criteria stipulate that participants are aged 75 or over and community-dwelling [15]. People with terminal cancer, life expectancy of 3 months or less and people in receipt of palliative care are excluded. Care or residential-home residents are not eligible to enrol onto the CARE75 + study. However, if a participant moves into one of these facilities after being enrolled, follow-up assessments are continued where possible. When recruited for the CARE75 + cohort participants gave informed consent for their data to be analysed by approved researchers. Approval for this study was granted by the CARE75 + Data Request Review Committee (DRRC) [12] in March 2021.

### Variables

The Nottingham Extended Activities of Daily Living (NEADL) scale was the primary outcome for this study [16]. NEADL scores were obtained from participants’ assessments conducted at baseline and at 12 and 24 month follow-up. The NEADL asks about the activities the respondent has completed within the last four weeks. These include 22 activities covering four dimensions: mobility [1–6], kitchen [7–11], domestic [12–16], and leisure [17–22]. The NEADL is used as an indication of independence [11, 17, 18] based on assessment of an individual’s ability to complete IADLs without help. Activities within each dimension are scored 0,1,2, or 3 for being unable, able with help, able alone but with difficulty, or able alone to perform an activity, respectively [10]. Scores for each activity are summed to give a total NEADL score between 0 and 66. Higher scores demonstrate greater independence [16].

The NEADL outcome measure was chosen for this study as it is used in current practice [19–21] and is interpreted as a direct measure of independence [16]. The measure has not been validated for use as a categorical variable; existing uses of the NEADL as a binary measure suggest that a score of ≤ 43 indicates dependence but lack evidence to support its use in this way [20, 22]. As a continuous variable the NEADL has good face validity and internal consistency (Cronbach’s alpha: 0.9) [10].

**Fig. 1:**
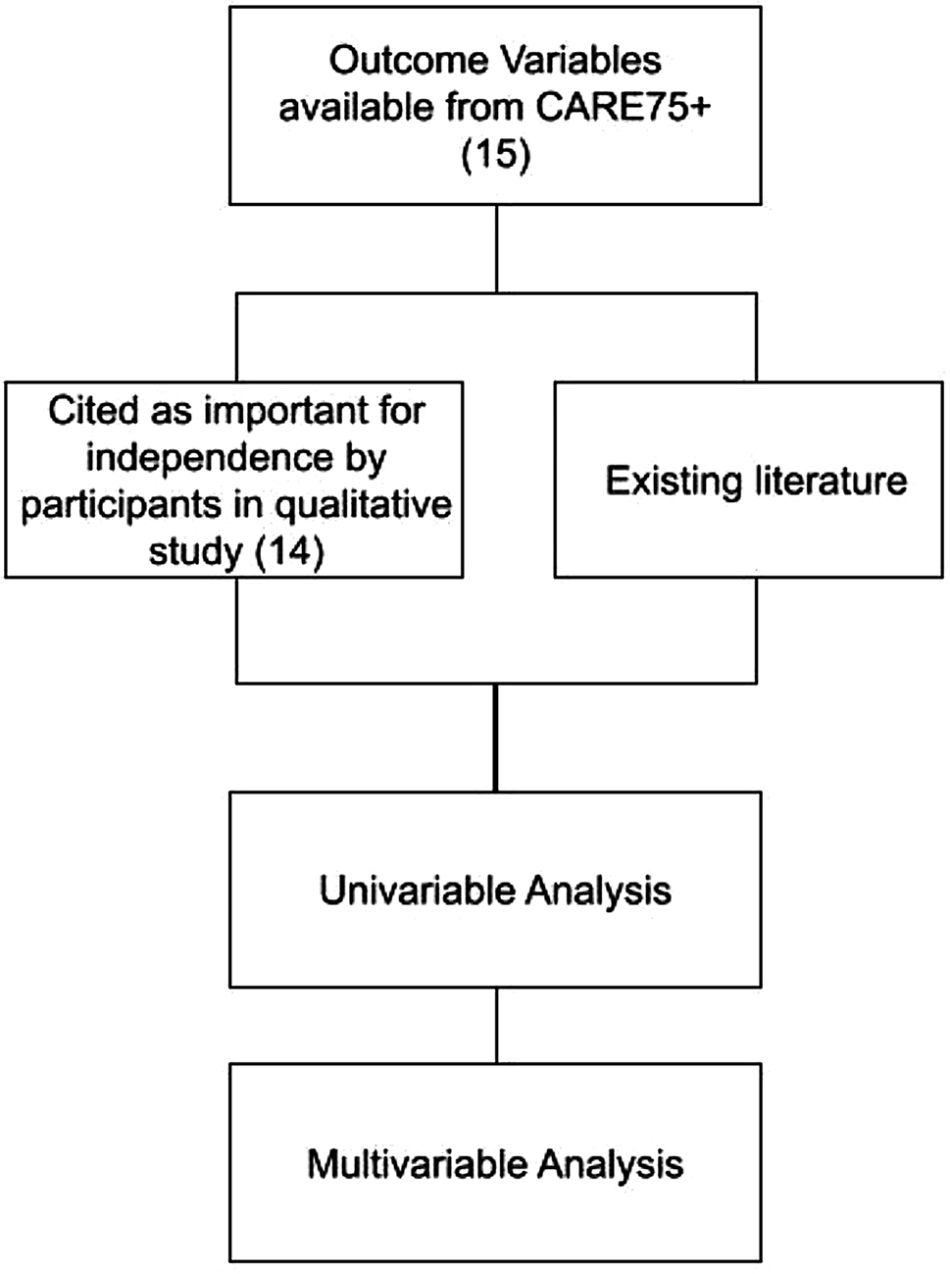
A diagram depicting the selection process for outcome variables

The pool of potential predictor variables comprised measures collected through CARE75 + baseline assessments. The selection of predictor variables from this pool was informed by a review of existing literature and an exploratory qualitative study [14] in which participants were asked about the most important facilitators of independence from their perspective. Selected variables were identified by the lead author and discussed with a second member of the research team with any disagreements on inclusion considered and resolved. The following variables were identified: sex [23–25], ethnicity [26] Age [21, 23–25, 27], Living Circumstances [14, 28], House Type [14, 27]; IMD (Index of Multiple Deprivation) [29]; Highest Qualification [23–24, 27]; number of family connections (children, grandchildren) [14, 24, 30–31]; Informal (unpaid) support [23]; recent use of care services [14]; equipment [32]; smoking status [12, 27]; alcohol consumption [12, 27]; sensory impairment [12, 27]; quality of life (SF-36) [25]; cognitive function (MoCA) [12, 25, 27]; comorbidity [12, 24]; extent of polypharmacy [12, 24, 33]; falls status [27]; grip strength [34]; frailty status [24, 29], depression [25, 27], resilience [14, 35], self-efficacy [36, 37] and baseline ability to complete instrumental (NEADL) [12] and basic (Barthel) activities of daily living [12]. A diagram illustrating the selection process is provided in Fig. 1. and further details are available in Appendix 1.

### Statistical analysis

Baseline characteristics of participants were summarised using means and standard deviations (SDs) for continuous variables and numbers and percentages for categorical variables. To refine the selection of candidate predictor variables and reduce the potential for overfitting of the multivariable model, univariable models were run separately for each predictor variable against the outcome variable. In the univariable models a threshold p-value of < 0.1 was used to determine whether variables should be included in the multivariable model. This relatively high threshold value was used to reduce the risk of omitting important variables whose predictive value was masked by lack of controlling of covariates in univariable models [38]. We used a p-value of < 0.05 as the threshold to identify statistically significant predictors of independence in the multivariable model [39]. Estimated effects with 95% confidence intervals (CIs) and p-values are reported for the univariable and multivariable analyses.

We conducted multiple linear regression using Stata 17 [40] to assess the association between potential predictor variables and NEADL score (continuous measure) at 12 and 24 months from baseline.

Linear regression assumes the presence of homoskedasticity, or equal variance of observations, and independence of predictor variables. To avoid the risk of generating unreliable results due to their violation, visual checks (using residual versus continuous predictor plots and a residual versus fitted values plot) were used to confirm that the assumptions were warranted in this study [41].

Potential correlation between independent variables was examined using correlation statistics, to identify and prevent problematic changes in the value of the regression coefficient due to correlation between predictor variables. Symptoms of multi-collinearity were checked by inspecting correlation coefficients and Variance Inflation Factors (VIF). A correlation coefficient of 0.80 or higher is generally considered “too high”, but may not be sensitive enough and ultimately the decision is made by the researcher [42]. In this study, if the correlation coefficient between two potential predictors was greater than or equal to 0.70, then only one of the potential predictors would be included in the model. The decision on which predictor to keep was decided based on the researcher’s knowledge of the field and determination of which was most pertinent to the concept being studied within the model. VIF scores quantify the change in variance incurred by inclusion of the corresponding variable in the model [43]. Scores of > 10 indicate a potential multicollinearity problem and a need to exclude corresponding variables from this study.

### Missing data

When large quantities of data are missing, complete case analysis is at increased risk of bias and reduced precision [44]. There is no established method for imputing missing values if individual items within the NEADL scale are missing and ad hoc solutions, such as ‘last observation carried forward’ or deletion methods are likely to incur bias [11, 45]. Multiple Imputation (MI) is an appropriate method for reducing this potential for bias and has been shown to improve the accuracy of results in similar studies [46]. MI using Predictive Mean Matching (PMM) with a k-nearest neighbour (knn) of 10 was used and 50 imputed datasets were generated [47]. PMM is an appropriate approach to MI when working with variables that are not normally distributed and when imputed values should not fall outside of the observed values, which is the case for our data [48]. Outcome variables at all time-points were included in the MI process, along with all of the potential predictor variables selected prior to refinement using univariable models. Variables with no missing data were included in the model as auxiliary variables to inform the imputation process [49].

## Results

### Sample characteristics

Data were obtained for all consenting participants in the CARE75 + study (*N* = 1277).

Table 1 shows the baseline characteristics for all 1277 participants and 674 complete cases. The mean age of participants was 84.61 years (SD 4.95). Just over half of the sample were female (*n* = 655, 51.29%) and most participants were from a White ethnic group (*n* = 1,198, 93.81%). Just over half of participants had no formal qualifications (*n* = 677, 53.01%) whilst 144 (11.28%) had a bachelor’s degree or higher. At baseline, participants had a mean NEADL score of 53.82 (SD 13.19).

**Table 1:** Baseline characteristics of participants

Baseline characteristic		Complete Cases *N* = 674	All Cases *N* = 1277
Sex	Male, n (%)	335 (49.70)	622 (48.71)
	Female, n (%)	339 (50.30)	655 (51.29)
	Missing, n (%)	0 (0.00)	0 (0.00)
Age	Mean (SD)	84.19 (4.55)	84.61 (4.95)
	Missing, n (%)	0 (0.00)	0 (0.00)
Ethnicity	White, n (%)	640 (94.96)	1,198 (93.81)
	Caribbean (Black or Mixed Black/White), n (%)	4 (0.59)	5 (0.39)
	Asian, n (%)	30 (4.45)	73 (5.72)
	Other, n (%)	0 (0)	1(0.08)
	Missing, n (%)	0 (0.00)	0 (0.00)
Qualifications	GCSE, n (%)	105 (15.58)	190 (15.24)
	HNC/HND, n (%)	46 (6.82)	89 (7.14)
	Diploma, n (%)	57 (8.46)	91 (7.30)
	AS and A Level, n (%)	34 (5.04)	56 (4.49)
	Bachelor’s degree, n (%)	48 (7.12)	100 (8.02)
	Postgraduate, n (%)	20 (2.97)	44 (3.53)
	No qualifications, n (%)	364 (54.01)	677 (54.29)
	Missing, n (%)	0 (0.00)	30 (2.35)
House-Type	Semi-detached, n (%)	192 (28.49)	359 (28.13)
	Bungalow, n (%)	162 (24.04)	303 (23.75)
	Detached house, n (%)	154 (22.85)	290 (22.73)
	Terraced house, n (%)	96 (14.24)	184 (14.42)
	Flat, n (%)	51 (7.57)	104 (8.15)
	Sheltered housing, n (%)	17 (2.52)	28 (2.19)
	Extra care housing, n (%)	2 (0.30)	8 (0.63)
	Missing, n (%)	0 (0.00)	1 (0.08)
Living Circumstances	Living with partner/spouse, n (%)	337(50.00)	624 (49.02)
	Living Alone, n (%)	274 (40.65)	507 (39.83)
	Living with Family, n (%)	63 (9.35)	142 (11.15)
	Missing, n (%)	0 (0.00)	4 (1.44)
Frequency of alcoholic beverage in last year	3–4 days a week or more, n (%)	222 (32.94)	383 (30.21)
	1–2 days a week, n (%)	136 (20.18)	254 (20.03)
	1–2 times a month, n (%)	60 (8.90)	110 (8.68)
	Every other month or less, n (%)	90 (13.35)	168 (13.25)
	Not at all in the last 12 months, n (%)	166 (24.63)	353 (27.84)
	Missing, n (%)	0 (0.00)	9 (0.70)
Current Smoker	Yes, n (%)	36 (5.34)	66 (5.19)
	No, n (%)	638 (94.66)	1,205 (94.81)
	Missing, n (%)	0 (0.00)	6 (0.47)
Difficulty hearing	No Difficulty, n (%)	519 (77.00)	904 (72.49)
	Some difficulty, n (%)	154 (22.85)	340 (27.27)
	Unable to hear, n (%)	1 (0.15)	3 (0.24)
	Missing, n (%)	0 (0.00)	30 (2.35)
Blind or Partially Sighted	Yes, n (%)	16 (2.37)	35 (2.89)
	No, n (%)	658 (97.63)	1,177 (97.11)
	Missing, n (%)	0 (0.00)	65 (5.09)
Total number of health conditions	Mean (SD)	3.79 (2.30)	3.87 (2.54)
	Missing, n (%)	0 (0.00)	0 (0.00)
Total number of prescribed medications	Mean (SD)	6.05 (3.99)	5.96 (4.16)
	Missing, n (%)	0 (0.00)	0 (0.00)
How many falls in last 12 months	Mean (SD)	2.01 (2.52)	0.62 (1.68)
	Missing, n (%)	0 (0.00)	16 (1.25)
Dominant Mean Grip Strength	Mean (SD)	20.35 (9.49)	20.04 (10.18)
	Missing, n (%)	0 (0.00)	74 (5.79)
Electronic Frailty Index	Mean (SD)	0.21 (0.12)	0.22 (0.12)
	Missing, n (%)	0 (0.00)	171
MoCA	Mean (SD)	24.97 (4.31)	24.73 (4.43)
	Missing, n (%)	0 (0.00)	72 (5.64)
Barthel	Mean (SD)	19.33 (1.51)	19.10 (2.06)
	Missing, n (%)	0 (0.00)	22 (13.39)
Depression	Not depressed, n (%)	592 (87.83)	1,083 (86.16)
	Depressed, n (%)	82 (12.17)	174 (13.84)
	Missing, n (%)	0 (0.00)	20 (1.57)
Resilience	Mean (SD)	3.84 (0.64)	3.81(0.65)
	Missing, n (%)	0 (0.00)	58 (4.54)
General Self Efficacy	Mean (SD)	3.31 (0.44)	3.26 (0.49)
	Missing, n (%)	0 (0.00)	29 (2.27)
NEADL	Mean (SD)	55.30 (11.09)	53.82 (13.19)
	Missing, n (%)	0 (0.00)	41 (3.21)

SD = standard deviation; GCSE = General Certificate of Secondary Education; HND/C = Higher National Diploma/Certificate; NEADL = Nottingham Extended Activities of Daily Living

There were 674 complete cases (participants) within our dataset at 12 months follow-up. Through MI, data were generated for the missing data of 606 participants. Distributions of the observed (674 complete cases), and completed (1277 cases after MI) data were assessed graphically to check the fit of the imputation model [50].

### Univariable models

The inclusion of predictors in the univariable models was predominantly based on what older people perceived to be important for their independence in an exploratory qualitative study [14]. The predictors: age, ethnicity, sex, and frailty, appeared to be taken for granted by participants in the qualitative study but were included as potential predictors of independence based on a review of existing literature [51]. The table in Appendix 1 provides a list of the selected variables and reasoning for their inclusion.

The estimated effects from separate univariable models and the main multivariable model, are shown in Table 2 for NEADL at 12 months from baseline. Table 3 shows the results from models fitted to NEADL at 24 months from baseline. Only three variables resulted in a p-value of ⋝0.1 in the univariable model and were therefore excluded from either the 12 or 24-month multivariable analyses or both. These were: the binary variable denoting whether a participant had received outpatient treatment in the last four weeks (excluded from both 12 (*p* = 0.221) and 24 (*p* = 0.473) month models), and the total number of people a participant could call on (excluded from the 12-month (*p* = 0.677) model but not the 24-month (*p* = 0.033) model). Smoking status was excluded from the 24-month (*p* = 0.303) but not the 12-month (*p* = 0.074) multivariable model. There was no indication that any of the identified variables should be excluded due to problematic levels of multi-collinearity.

**Table 2:** Estimated effects of baseline predictor variables on NEADL score at 12 months

Outcome Variable: NEADL at 12 months		Univariable Analyses			Multivariable Analysis Adj. *R*-squared: 0.721		
**Main Variable**	**Categories**	**Estimated effect**	**95% CI**	**p-value**	**Estimated effect**	**95% CI**	**p-value**
Sex	Female	ref		0.037	ref		0.864
	Male	1.62	0.10 to 3.15		-0.11	-1.36 to 1.14	
Age		-0.98	-1.13 to -0.83	< 0.001	-0.20	-0.32 to -0.07	0.002
Ethnicity							
	White	ref		< 0.001	ref		0.053
	Black Caribbean and Mixed Black Caribbean/White	-11.71	-22.67 to -0.75		-1.96	-8.32 to 4.41	
	Asian	-22.89	-25.94 to -19.83		-4.09	-6.93 to -1.26	
	Other	-4.85	-33.29 to 23.59		2.45	-17.58 to 22.49	
House-Type							
	Semi-detached	ref		< 0.001	ref		0.174
	Bungalow	-0.54	-2.66 to 1.58		-0.51	-1.80 to 0.79	
	Detached house	2.29	0.16 to 4.43		-0.58	-1.87 to 0.70	
	Terraced house	-5.20	-7.64 to -2.76		0.46	-0.99 to 1.91	
	Flat	-0.07	-3.09 to 2.95		0.07	-1.69 to 1.83	
	Sheltered housing	-10.21	-15.71 to -4.71		-4.03	-7.62 to -0.44	
	Extra care housing	-12.46	-21.99 to -2.94		-2.98	-8.43 to 2.47	
Living Circumstances							
	Living with partner/spouse	ref		< 0.001	ref		0.009
	Living Alone	-1.07	-2.62 to 0.48		1.41	0.34 to 2.48	
	Living with Family	-15.06	-17.49 to -12.62		-0.76	-2.48 to 0.96	
Qualifications							
	No qualifications	ref		< 0.001	ref		0.851
	GCSE	7.97	5.82 to 10.12		0.74	-0.62 to 2.11	
	HNC/HND	7.46	4.50 to 10.43		0.78	-1.03 to 2.58	
	Diploma	7.39	4.47 to 10.32		1.07	-0.63 to 2.76	
	AS and A Level	7.48	3.86 to 11.11		0.13	-2.00 to 2.26	
	Bachelor’s Degree	6.84	4.03 to 9.66		0.07	-1.69 to 1.82	
	Postgraduate	6.26	2.11 to 10.41		0.20	-2.30 to 2.70	
IMD		1.48	1.21 to 1.75	< 0.001	0.09	-0.09 to 0.28	0.328
Total no. of children		-1.26	-1.78 to -0.74	< 0.001	0.02	-0.42 to 0.45	0.943
Total no. of grandchildren		-0.28	-0.53 to -0.04	0.022	0.07	-0.13 to 0.27	0.512
Total Number of contacts to call on		0.12	-0.45 to 0.69	0.677			
Hours of informal* support in the last 4 weeks		-0.12	-0.13 to -0.10	< 0.001	-0.02	-0.03 to -0.01	0.002
Hours of formal support in the last 4 weeks		-0.35	-0.45 to -0.25	< 0.001	0.05	-0.02 to 0.12	0.162
GP visit in the last 4 weeks	No	ref		0.005	ref		0.502
	Yes	-2.39	-4.04 to -0.73		0.32	-0.62 to 1.26	
Outpatient visit in the last 4 weeks	No	ref		0.221	ref		
	Yes	-1.18	-3.06 to 0.71				
Equipment**		-2.77	-3.08 to -2.46	< 0.001	-0.06	-0.35 to 0.23	0.674
Current Smoker	No	ref		0.074	ref		0.112
	Yes	-3.25	-6.82 to 0.32		-1.78	-3.98 to 0.42	
Alcohol Consumption over the last year							
	None	ref		< 0.001	ref		0.408
	≥ 3–4 days a week	10.65	8.73 to 12.56		1.14	-0.16 to 2.44	
	1–2 days a week	10.41	8.28 to 12.54		1.20	-0.18 to 2.58	
	1–2 times a month	8.90	6.05 to 11.75		0.52	-1.26 to 2.31	
	≤ Once a month	7.21	4.75 to 9.67		0.92	-0.64 to 2.47	
Difficulty Hearing							
	No difficulty	ref		< 0.001	ref		0.982
	Some difficulty	-6.91	-8.66 to -5.17		0.12	-0.97 to 1.21	
	Unable to hear	-7.94	-27.52 to 11.65		-0.55	-15.16 to 14.06	
Registered Blind or Partially Sighted	No	ref		< 0.001	ref		0.009
	Yes	-15.33	-20.25 to -10.42		-4.05	-7.08 to -1.02	
Total number of health conditions		-1.29	-1.59 to -0.99	< 0.001	0.03	-0.19 to 0.25	0.800
Total number of prescribed medications		-0.74	-0.93 to -0.56	< 0.001	0.02	-0.11 to 0.16	0.730
How many falls in last year		-1.10	-1.56 to -0.65	< 0.001	0.27	-0.01 to 0.54	0.051
Dominant Mean Grip Strength		0.48	0.41 to 0.55	< 0.001	0.02	-0.05 to 0.09	0.583
Electronic Frailty Index		-47.63	-53.83 to -41.43	< 0.001	-9.57	-15.21 to -3.93	0.001
MoCA		1.62	1.47 to 1.76	< 0.001	0.19	0.04 to 0.33	0.010
SF-36 MCS		0.32	0.23 to 0.42	< 0.001	-0.02	-0.09 to 0.05	0.607
SF-36 PCS		0.61	0.55 to 0.67	< 0.001	0.07	0.01 to 0.12	0.014
Barthel		3.79	3.46 to 4.11	< 0.001	0.61	0.26 to 0.96	0.001
Depression	Not depressed	ref		< 0.001	ref		< 0.001
	Depressed	-16.75	-18.86 to -14.64		-3.17	-4.87 to -1.48	
Resilience		6.18	4.98 to 7.37	< 0.001	0.16	-0.74 to 1.07	0.721
General Self Efficacy		9.41	7.89 to 10.92	< 0.001	0.12	-0.99 to 1.24	0.828
Baseline NEADL		0.85	0.82 to 0.89	< 0.001	0.54	0.47 to 0.60	< 0.001

ref = reference variable; CI = Confidence Interval; GCSE = General Certificate of Secondary Education; HND/C = Higher National Diploma/Certificate; NEADL = Nottingham Extended Activities of Daily Living; IMD = Index of Multiple Deprivation; MCS = Mental Component Scale; PCS = Physical Component Scale

*Informal support is defined as unpaid/voluntary support

**Equipment is determined by the number of pieces of equipment a person has in their home

**Table 3:** Estimated effects of baseline predictor variables on NEADL score at 24 months

Outcome Variable: NEADL at 24 months		Univariable Analyses			Multivariable Analysis Adj. *R*-squared: 0.657		
**Main Variable**	**Categories**	**Estimated effect**	**95% CI**	**p-value**	**Estimated effect**	**95% CI**	**p-value**
Sex	Female	ref		0.005	ref		0.753
	Male	2.58	0.78 to 4.38		0.28	-1.45 to 2.00	
Age		-1.15	-1.32 to -0.97	< 0.001	-0.37	-0.55 to -0.19	< 0.001
Ethnicity							
	White	ref		< 0.001	ref		0.446
	Black Caribbean and Mixed Black Caribbean/White	-9.32	-21.53 to 2.89		-1.11	-8.91 to 6.68	
	Asian	-18.79	-22.31 to -15.26		0.71	-2.97 to 4.38	
	Other	9.08	-18.17 to 36.34		14.86	-2.53 to 32.24	
House-Type							
	Semi-detached	ref		< 0.001	ref		0.617
	Bungalow	-1.81	-4.17 to 0.56		-1.30	-2.95 to 0.35	
	Detached house	1.99	-0.39 to 4.38		-0.73	-2.42 o 0.95	
	Terraced house	-5.06	-7.88 to -2.25		-0.47	-2.48 to 1.54	
	Flat	-2.07	-5.55 to 1.40		-1.25	-3.71 to 1.21	
	Sheltered housing	-8.99	-15.32 to -2.67		-1.85	-6.36 to 2.67	
	Extra care housing	-15.85	-26.47 to -5.22		-5.50	-12.86 to 1.85	
Living Circumstances							
	Living with partner/spouse	ref		< 0.001	ref		0.028
	Living Alone	-1.87	-3.73 to -0.02		1.98	0.46 to 3.49	
	Living with Family	-13.26	-16.07 to -10.46		-0.003	-2.49 to 2.48	
Qualifications							
	No qualifications	ref		< 0.001	ref		0.724
	GCSE	7.77	5.35 to 10.19		0.42	-1.35 to 2.18	
	HNC/HND	7.67	4.27 to 11.06		0.82	-1.63 to 3.26	
	Diploma	6.88	3.54 to 10.23		1.00	-1.36 to 3.36	
	AS and A Level	5.64	1.52 to 9.75		-1.93	-4.76 to 0.89	
	Bachelor’s Degree	6.71	3.45 to 9.96		-0.38	-2.74 to 1.99	
	Postgraduate	6.36	1.60 to 11.11		0.40	-2.99 to 3.79	
IMD		1.34	1.03 to 1.65	< 0.001	0.09	-0.16 to 0.33	0.482
Total no. of children		-0.86	-1.48 to -0.23	0.007	0.37	-0.28 to 1.01	0.261
Total no. of grandchildren		-0.25	-0.53 to 0.04	0.090	-0.01	-0.28 to 0.26	0.931
Total Number of contacts to call on		0.68	0.06 to 1.30	0.033	0.15	-0.29 to 0.59	0.510
Hours of informal* support in the last 4 weeks		-0.11	-0.13 to -0.09	< 0.001	-0.01	-0.03 to < 0.01	0.080
Hours of formal support in the last 4 weeks		-0.36	-0.47 to -0.24	< 0.001	0.06	-0.03 to 0.16	0.196
GP visit in the last 4 weeks	No	ref		0.001	ref		0.444
	Yes	-3.43	-5.39 to -1.48		-0.54	-1.92 to 0.85	
Outpatient visit in the last 4 weeks	No	ref					
	Yes	-0.77	-2.87 to 1.33	0.473			
Equipment**		-3.10	-3.47 to -2.73	< 0.001	-0.23	-0.62 to 0.16	0.244
Current Smoker	No	ref		0.303			
	Yes	-2.17	-6.29 to 1.96				
Alcohol Consumption over the last year							
	None	ref		< 0.001			0.002
	≥ 3–4 days a week	11.21	9.01 to 13.41		2.21	0.49 to 3.94	
	1–2 days a week	10.93	8.53 to 13.33		2.40	0.55 to 4.25	
	1–2 times a month	11.70	8.52 to 14.87		4.20	1.89 to 6.52	
	≤ Once a month	8.14	5.34 to 10.94		3.11	1.05 to 5.17	
Difficulty Hearing							
	No difficulty	ref		< 0.001	ref		0.170
	Some difficulty	-6.75	-8.74 to -4.75		0.98	-0.53 to 2.49	
	Unable to hear	-18.77	-39.49 to 1.96		-11.12	-27.83 to 5.58	
Registered Blind or Partially Sighted	No	ref		< 0.001	ref		0.135
	Yes	-15.26	-20.75 to -9.77		-2.90	-6.71 to 0.91	
Total number of health conditions		-1.59	-1.95 to -1.23	< 0.001	-0.18	-0.51 to 0.15	0.274
Total number of prescribed medications		-0.81	-1.03 to -0.60	< 0.001	0.07	-0.12 to 0.27	0.466
How many falls in last year		-1.53	-2.03 to -1.02	< 0.001	-0.10	-0.45 to 0.25	0.567
Dominant Mean Grip Strength		0.54	0.45 to 0.62	< 0.001	0.05	-0.03 to 0.14	0.216
Electronic Frailty Index		-52.88	-59.83 to -45.93	< 0.001	-9.09	-17.16 to -1.03	0.027
MoCA		1.65	1.48 to 1.82	< 0.001	0.40	0.17 to 0.62	0.001
SF-36 MCS		0.35	0.25 to 0.46	< 0.001	0.00	-0.09 to 0.10	0.965
SF-36 PCS		0.66	0.60 to 0.73	< 0.001	0.11	0.04 to 0.18	0.002
Barthel		3.85	3.45 to 4.24	< 0.001	0.65	0.16 to 1.14	0.010
Depression	Not depressed	ref		< 0.001	ref		0.003
	Depressed	-17.89	-20.27 to -15.50		-3.49	-5.81 to -1.18	
Resilience		6.62	5.30 to 7.93	< 0.001	0.07	-1.09 to 1.23	0.901
General Self Efficacy		9.89	8.18 to 11.60	< 0.001	0.51	-0.98 to 2.00	0.501
Baseline NEADL		0.83	0.78 to 0.88	< 0.001	0.42	0.32 to 0.52	< 0.001

ref = reference variable; CI = Confidence Interval;; GCSE = General Certificate of Secondary Education; HND/C = Higher National Diploma/Certificate; NEADL = Nottingham Extended Activities of Daily Living; IMD = Index of Multiple Deprivation; MCS = Mental Component Scale; PCS = Physical Component Scale

*Informal support is defined as unpaid/voluntary support

**Equipment is determined by the number of pieces of equipment a person has in their home

### Multivariable models

#### Predictors of NEADL at 12-months

In the multivariable model (see Table 2), being older, receiving higher levels of unpaid informal help, being registered blind or partially sighted, being frail and having depressive symptoms were associated (p-value < 0.05) with a lower NEADL score suggesting that participants with higher scores on these predictors at baseline would have lower independence at 12 months from baseline than those with lower baseline scores for these predictors. Living alone, having higher cognitive function (MoCA score), and greater physical function (SF-36 Physical Component Scale, Barthel index and baseline NEADL) were associated with higher independence at 12 months with p-value < 0.05.

#### Predictors at 24-months

In contrast to the results at 12-months, at 24-months (see Table 3), being registered as blind or partially sighted (*p* = 0.135), and level of informal support (*p* = 0.080) were not significant predictors of independence. Age, baseline NEADL score, living alone, frailty, cognitive function, physical function and depressive symptoms were significant, and the direction of prediction was the same as in the 12-month model. Alcohol consumption was a significant predictor of independence at 24-months but not at 12-months. Compared to not drinking alcohol at all, participants across all alcohol consumption groups were predicted to have higher independence at 24-months (*p* = 0.002).

## Discussion

This study identified predictors of functional independence from a broad range of variables. The inclusion of older people’s views, in addition to reviewing the literature, to inform the inclusion of variables added a novel breadth to the research. This approach was augmented by the inclusion of models with NEADL as the outcome at 12- and 24-months post baseline, enabling exploration of variables’ stability as predictors over time. The relatively large number of variables with p-value < 0.1 in the univariable models demonstrates the wide range of influences on independence when it is measured by ability to perform activities of daily living. The breadth of variables contributed a key strength of this study as, by acting as covariates within the model, significant predictors were determined whilst accounting for the inherent complexity of independence.

The importance of frailty and depression in prediction of independence over time is consistent with existing literature. A systematic review by Kojima et al. demonstrated the relationship between frailty and disability, and a recent paper by Coventry et al. demonstrated, not only independent associations, but also a moderating relationship between depression and frailty on independence [52, 53]. Furthering the understanding provided in these, and other existing studies, this study demonstrates how the predictive value of frailty and depression persists when both extra-individual (e.g., medication use, social support, attendance at health services) and intra-individual (e.g., self-efficacy, resilience, perceived health) factors are accounted for [52, 54]. Notably, the results demonstrated that lower cognitive impairment predicted a significantly higher independence score independently of depression or frailty, a relationship that earlier studies suggested required further investigation [52].

Both depression and frailty encompass a multitude of symptoms and presentations, many of which overlap making it difficult to disentangle the unique mechanisms underpinning their impact on independence [55, 56]. Refining understanding of which dimensions of frailty and depression are most potent for the prediction of independence could increase the specificity of targeted interventions [55]. Shedding some light on the relationship between depression, frailty and their sub-dimensions we have shown that, though important to older people qualitatively, attributes such as resilience and self-efficacy were not significant predictors of independence when a wide range of covariates were accounted for in the multivariable model. The contrast between this finding and existing literature [57, 58] may indicate that resilience and self-efficacy impact independence only through their contribution to other predictors (e.g. depression) rather than independently. Another explanation for the difference in results of this study may be the efficacy of outcome measures used.

Our findings support the need for further work to focus on the specific dimensions of depression and frailty and the context in which they contribute to independence [55].

The estimated effects of assessments of basic (Barthel) and extended (baseline NEADL) functional abilities as well as physical components of quality of life were statistically significant but small. For every 1 point increase on these assessments at baseline an increase of independence of less than two points could be predicted at both 12-months and 24-months from baseline. Therefore, In comparison to the predictors depression (estimated effect:-3.17; CI: -4.87 to -1.48) and frailty (estimated effect: -9.57; CI: -15.21 to -3.93), baseline functional ability predicts relatively less change in functional ability over time than depression and frailty do. This result aligns with findings from studies showing that engagement in specific tasks or activities is determined by more than the physical capacity to do it. Psychosocial aspects, such as: the value attributed to a given task [59, 60], motivation [61] and confidence [62] to achieve it are all important contributors to function which would be hindered by the experience of depression. Assessments of functional ability are insensitive to individual efforts of adaptation [63]. Characterised by a reduced resistance to stress [64], increased frailty reduces the resources that a person has to make adaptation feasible which may explain the much starker predictions of decline associated with this variable. These results challenge the efficacy of interventions for independence and rehabilitation that focus on physical and functional aspects alone and support the need for more complex support that addresses the multi-faceted and inter-related predictors of frailty and mental health.

Living circumstances, determined by who a person lived with, were a significant predictor of independence in both analyses. Living alone predicted greater independence at 12- and 24-months compared with living with a spouse or family. The NEADL scale is scored based on what someone ‘does do’ rather than ‘can do’ on their own and without help [10]. Therefore regardless of a person’s ability, receiving help with a task is penalised in the scoring system [16]. Living with other people means that sources of help are more readily available whilst living alone places greater obligation on a person to complete tasks for themselves [65]. This may explain why participants who were categorised as ‘living alone’ had, on average, higher NEADL scores, demonstrating a greater semblance of independence.

Having no other option than to complete tasks themselves the difference in score for people living alone may reflect a decrease in deconditioning that can occur from lack of involvement in a given task [66]. However, although receiving help is presented as a deficit in the scoring system, it may be part of a reciprocal and mutually beneficial relationship of interdependence [67]. This may be preferable as it allows for the reservation of personal energy to be used for activities that hold greater value to the individual [1, 68].

Further, the benefit of social connection offered by an interdependent relationship may have other important health benefits such as reduced mortality and biological manifestations of ageing [30]. However, living circumstances may only be a crude indicator of social connection since it is not just the presence but the quality of a relationship that is important for health and wellbeing outcomes [24, 30, 31]. For example, someone living alone who regularly goes out to visit or share experiences with friends may in fact be more socially connected than someone whose only regular social contact is the spouse or family member they live with. The direction of the relationship between living circumstances and independence found in these results warrants further exploration to understand to what extent living alone impacts independence over and above the impact that a propensity for independence has on the determination of living circumstance. Further, the beneficial impact of living alone on independence identified in these results need to be evaluated in the wider social context in which living alone may have simultaneous negative impacts on wellbeing, loneliness and isolation [69].

The finding that alcohol consumption was a significant predictor of independence at 24-months but not at 12-months was unexpected and seems incongruent with the known impacts of alcohol consumption such as increased morbidity and falls [70].Whilst there is some evidence that characteristics associated with low-moderate drinkers may have protective effects for mortality [70, 71], there is little evidence of this association for more excessive patterns of drinking. The finding was not explained by participants’ responses in the preceding qualitative study. Further exploration of the interrelated relationships contributing to the impact of alcohol consumption over time warrants further exploration but was beyond the scope of this study.

Strengths and Limitations.

A key strength of this study was that we included a wide variety of variables that were important to older people as well as identified through existing literature. This reduced the risk of unreliability of the outcome due to unmeasured variables and generated novel understanding about the performance of predictors in the context of a wide range of covariates. A further strength of this study was the use of data from the CARE75 + cohort. Strategically designed to improve diversity and rate of recruitment the CARE75 + cohort provided access to high quality data from a large cohort of older people from varied urban and rural locations across the country [72, 73].

Missing data were accounted for using a method of MI selected based on its suitability to the data available. MI relies on estimation of missing results, which may not be as accurate as complete cases, but is a principled and robust method of accounting for missing data [50]. The risk that controlling for a wide range of variables had the potential to introduce bias into the results through over-fitting of the model was reduced by the large cohort sample and by ensuring that there were sufficient observations per predictor variable included in the model [74]. Despite work by the CARE75 + team to ensure a representative sample, the involvement of participants from ethnic groups that were not white was low and, as acknowledged, limited the conclusions that could be drawn from this aspect of the research [72].

There is a lack of robust evidence to justify what change in NEADL score would correspond with a clinically meaningful change for an individual older person [75]. In a study conducted with people affected by Parkinson’s, a change of 2.5 or more NEADL points was deemed to indicate a clinically meaningful difference in independence [76]. However, it is unclear how the value of 2.5 was determined and whether it would apply for older people not affected by Parkinson’s. This makes it difficult to draw conclusions about whether the changes seen are sufficient to have a meaningful impact for person-centred or clinical outcomes and highlights an area for future research. A change in NEADL of just 1–2 points, which could be the difference between relying on someone’s help to wash oneself and being able to complete this task alone [13] has the potential to make a meaningful difference to an individual.

The proportion of male to female participants in our sample differs from that of the general population. This may reflect the efficacy of Trial Within Cohort Studies [73] to be more inclusive in recruitment than traditional research studies. It is important that research findings are applicable to a broad range of the population experiencing the condition or phenomenon under study [77]. Although males represent a smaller proportion of the population of people aged 75+, they are also less likely to be involved in research than females of the same age [78] and therefore their perspectives and experiences tend to be under-represented in research. The high value placed on maintaining independence is evident for both males [79] and females [80] aged 75 + and therefore it is important that the predictors identified are likely to be relevant to both sexes.

This study relied on quantitative data collected within the CARE75 + cohort study. The use of secondary data had pragmatic and ethical benefits by reducing the researcher and participant burden that would have been incurred by additional data collection. However, a limitation of this approach was that the variables available did not always align with the purpose of this study. For example, for the variables ‘hours of informal/formal help received’ the type of support offered is not specified and may or may not include support with completion of I/ADLs.

Implications for Future Research, Practice or Policy.

The results of this study provide policy-makers and providers of evidence-based practice with important understanding of the conditions and characteristics that may make some older people more susceptible to changes in their functional independence. The negative impact of depression and frailty on function in daily activities suggests that policy and practice to promote independence should have a focus on improving mental as well as physical health. Refining understanding about the mechanisms underpinning their prediction of independence could help to better target services to improve independence in older age.

## Electronic supplementary material

Below is the link to the electronic supplementary material.

Supplementary Material 1

## Data Availability

The data that support the findings of this study were made available through Bradford Teaching Hospitals NHS Foundation Trust. CARE75+ is led by the Academic Unit for Ageing and Stroke Research, University of Leeds, based at the Bradford Institute for Health Research, Bradford Teaching Hospitals NHS Foundation Trust. The funding for CARE75+ is provided by the Yorkshire and Humber Applied Research Collaborations. Restrictions apply to the availability of these data, which were used under license for the current study, and so are not publicly available. Data are however available from the authors upon reasonable request and with permission of Bradford Teaching Hospitals NHS Foundation Trust.

## Abbreviations

NEADL: Nottingham Extended Activities of Daily Living
ADLs: Activities of Daily Living
SD: standard deviation
CARE75 +: Community Ageing REsearch 75+
DRRC: Data Request Review Committee
CI: Confidence Interval
MI: Multiple Imputation
PMM: Predictive Mean Matching
knn: k-nearest neighbour
MoCA: Montreal Cognitive Assessment
CI: Confidence Interval
IMD: Index of Multiple Deprivation
MCS: Mental Component Scale
PCS: Physical Component Scale
