# Supplementary material for: Predictors of independence in older people: A longitudinal, population-based study using the CARE75 + cohort
Source: BMC Geriatr. 2025 Apr 28;25:288. doi: 10.1186/s12877-025-05927-4 (PMC12036217; doi:10.1186/s12877-025-05927-4)
Supplement: Supplementary file 1 — Supplementary Material 1 [file 12877_2025_5927_MOESM1_ESM.docx]

## Appendix 1

| **Variable** | **Details** | **Reason for Selection** | | | | | |
| --- | --- | --- | --- | --- | --- | --- | --- |
|  |  | **Key -** Variable selected based on: | | | | | |
|  |  |  | Existing literature alone |  | A mixture of qualitative responses and literature |  | Qualitative responses primarily |
| Sex | Dichotomous variable male or female | Male and female participants may differ in characteristics and values which may influence how they experience independence. A difference was perceived during interviews and sex has been shown to predict variance in ADL/IADL ability in older people (23-25) | | | | | |
| Ethnicity | Self-reported and separated into the categories: White; Black Caribbean and Mixed Black Caribbean/White; Asian; Other | A possible confounder because of cultural differences in the value of independence - it was not something that was brought up by the participants but is recognised as a standard confounder in many studies. (26) | | | | | |
| Age | Chronological age | Increased chronological age is associated with increased risk of physiological dysfunction and is included as a confounding variable based on existing literature.(21,23-25,27) | | | | | |
| Living Circumstances | Who the participant lives with.  Categorical options: lives alone, lives with partner/spouse, lives with family | Participants talked about how living with someone else could have implications on independence due to altered freedoms and availability of support.(14, 28) | | | | | |
| House Type | Categorical options: Bungalow, Semi-detached house, Detached house, Terraced house, Flat, Sheltered housing, Extra care housing | A couple of participants talked about how the fact that they lived in a bungalow or flat might help or hinder their independence. (14, 27) | | | | | |
| IMD | Indices of Deprivation derived from national data based on participant post code. Values from 1-10 with 10 indicating a person living within one of the 10 least deprived areas and 1 indicating living in one of the 10% least deprived areas. | Finance was brought up as a potential barrier or facilitator to independence by providing the means to engage in activities and access transport etc. Having sufficient financial resources may decrease the risk of becoming ADL dependent (29). | | | | | |
| Highest Qualification | Highest educational qualification attained Categorical options based on self-report:  No qualifications; GCSE; HNS/NHD; Diploma; AS and A Level; Bachelor’s Degree; Postgraduate | Education level is a recognised confounder. (23-24, 27) | | | | | |
| Children | How many children/grandchildren a person has | Strength and diversity of support networks have been identified as confounding factors in previous studies and the ability to call on help when needed was talked about in participants’ understandings of independence. (14, 23-24, 30-31) | | | | | |
| Grandchildren |  |  |  |  |  |  |  |
| Informal Support | No. of hours of unpaid support received per week |  |  |  |  |  |  |
| Recent GP visit | Visit within the last 4 weeks. Binary variable Yes/No | Several participants (14) talked with pride about the fact that they had not visited their GP or needed to use healthcare services. This variable provides insight into healthcare usage and facilitates exploration of whether avoidance of formal support is a predictor of independence. | | | | | |
| Recent Outpatient visit |  |  |  |  |  |  |  |
| Equipment | The number of listed items in a person’s home: Grab Rails, Toilet Seat, Bath Seat, Recliner Chair, Bed Rail, Bed Risers, Hoist, Helping Hand, Key Safe, Pendant Alarm, Stair Lift, Outside Step, Wheelchair | Most participants did not use equipment or home adaptations, but some did mention the use of pendant alarms and key-safes which provided reassurance for independence. Other bits of equipment were often perceived as making you old (and therefore less independent) too quickly so it would be interesting to see whether there is any difference in independence trajectory between different bits of equipment. Equipment is also prescribed on the basis that it should help people to maintain independence so would provide useful insight. (32) | | | | | |
| Current Smoker | Binary options Yes/No | Some participants related their good health, which helped them to be independent, to lifestyle factors such as not drinking and not smoking. These are also potentially modifiable factors making them clinically useful, and have been linked to independence in existing studies. (12, 27) | | | | | |
| Current alcohol consumption | Frequency of alcohol consumption in the last year from the options:  None; ≥3-4 days a week; 1-2 days a week; 1-2 times a month; ≤ Once a month |  |  |  |  |  |  |
| Blind or partially sighted | Registered blind or partially-sighted  Binary outcome – yes or no | None of the interviewed participants were registered blind or partially sighted, however, poor sight was a reason given by one potential participant for declining to take part in the interview study. Poor hearing was related to independence in some interviews because it could be isolating and increase dependence on a spouse or partner. (12, 27) | | | | | |
| Difficulty Hearing | Determined by assessor. Categorical options of: No difficulty; some difficulty; no hearing at all |  |  |  |  |  |  |
| SF-36 | Score out of 100 from the SF-36 Patient Reported Outcome Measure for quality of life | The SF-36 is a measure of quality of life and asks questions about ability to engage in purposeful activities, subjective health, mood, and pain. Each of these topics were spontaneously brought into conversations by participants in the qualitative study. (25) | | | | | |
| MoCA | Montreal Cognitive Assessment.  Scored 0-30. | The overall MoCA score gives an indicator level of cognitive function. Loss of cognitive abilities was definitely seen by participants as a contributor to becoming less independent. (12, 25, 27) | | | | | |
| Total number of comorbidities | Total number of diseases from GP record (excluding osteoporosis and fracture) | Participants mostly voiced a feeling of being 'lucky' to have their health and believed that it would be much harder to remain independent if their health was disrupted. Those that they considered more dependent were often people with a health condition. Comorbidity is a common confounder or exclusion criteria in studies so should be accounted for.(12, 24) | | | | | |
| Medication count | No. of prescribed medications | Many participants were on medications, but it was only said in passing and not something that they described as important to independence. Polypharmacy is a big issue in care of older people. (12, 24, 33) | | | | | |
| How many falls | Self-reported number of falls within the last year | Few participants talked about falls, but for those who did talk about them, either through own or other’s experience, they were often associated with considerable disruption to independence. (12, 27)) | | | | | |
| NEADL | Nottingham Extended Activities of Daily Living scale. Scored 0-66. Higher scores represent greater independence | Primary dependent outcome of interest (12) | | | | | |
| Barthel | Patient reported outcome measuring ability in basic activities of daily living. Total possible scores range from 0 – 20. Lower scores indicate greater disability | Participants’ talked about the importance of looking after themselves for independence, which included management of these fundamental activities of daily living. (12) | | | | | |
| Grip Strength | Measured by a dynamometer from dominant hand | A key marker of frailty and physical capacity (34). A useful tool in practice so it would be good to see how it compares to participant suggested variables. | | | | | |
| EFI | Electronic Frailty Index | Frailty can be linked to independence in practice and has the potential to be a strong confounder (24, 29). Participants did not use the word frail, but those that they considered more dependent had many of the traits associated with frailty. | | | | | |
| Geriatric Depression Score | A 15 item scale designed to aid screening for depression in older patients. Scores ≥5 suggest depression | Depression was clearly linked with dependence in several participant’s minds and in the literature. (12, 25, 27) | | | | | |
| Brief Resilience Scale | A patient-reported outcome to assess a person’s ability to ‘bounce back’ following an adverse event. Higher scores indicate greater resilience. | The qualitative study found that characteristics that related to resilience were very important for independence. (14, 35) | | | | | |
| Self-Efficacy | Measured by the General Self Efficacy scale. Higher scores indicate greater self-efficacy. | The importance of confidence and related factors for independence was brought up by several participants and was regarded as a predictive factor.(36-37) | | | | | |
